# Drugs affecting the renin-angiotensin system and survival from cancer: a population based study of breast, colorectal and prostate cancer patient cohorts

**DOI:** 10.1186/1741-7015-12-28

**Published:** 2014-02-13

**Authors:** Chris R Cardwell, Úna C Mc Menamin, Blánaid M Hicks, Carmel Hughes, Marie M Cantwell, Liam J Murray

**Affiliations:** 1 Cancer Epidemiology and Health Services Research Group, Centre for Public Health, Queen’s University Belfast, Belfast, Northern Ireland; 2 School of Pharmacy, Queen’s University Belfast, Belfast, Northern Ireland

**Keywords:** Colorectal cancer, Breast cancer, Prostate cancer, Mortality, Angiotensin-converting enzyme inhibitors and angiotensin II receptor blockers

## Abstract

**Background:**

Angiotensin-converting enzyme inhibitors (ACEIs) and angiotensin II receptor blockers (ARBs) are commonly prescribed to the growing number of cancer patients (more than two million in the UK alone) often to treat hypertension. However, increased fatal cancer in ARB users in a randomized trial and increased breast cancer recurrence rates in ACEI users in a recent observational study have raised concerns about their safety in cancer patients. We investigated whether ACEI or ARB use after breast, colorectal or prostate cancer diagnosis was associated with increased risk of cancer-specific mortality.

**Methods:**

Population-based cohorts of 9,814 breast, 4,762 colorectal and 6,339 prostate cancer patients newly diagnosed from 1998 to 2006 were identified in the UK Clinical Practice Research Datalink and confirmed by cancer registry linkage. Cancer-specific and all-cause mortality were identified from Office of National Statistics mortality data in 2011 (allowing up to 13 years of follow-up). A nested case–control analysis was conducted to compare ACEI/ARB use (from general practitioner prescription records) in cancer patients dying from cancer with up to five controls (not dying from cancer). Conditional logistic regression estimated the risk of cancer-specific, and all-cause, death in ACEI/ARB users compared with non-users.

**Results:**

The main analysis included 1,435 breast, 1,511 colorectal and 1,184 prostate cancer-specific deaths (and 7,106 breast, 7,291 colorectal and 5,849 prostate cancer controls). There was no increase in cancer-specific mortality in patients using ARBs after diagnosis of breast (adjusted odds ratio (OR) = 1.06 95% confidence interval (CI) 0.84, 1.35), colorectal (adjusted OR = 0.82 95% CI 0.64, 1.07) or prostate cancer (adjusted OR = 0.79 95% CI 0.61, 1.03). There was also no evidence of increases in cancer-specific mortality with ACEI use for breast (adjusted OR = 1.06 95% CI 0.89, 1.27), colorectal (adjusted OR = 0.78 95% CI 0.66, 0.92) or prostate cancer (adjusted OR = 0.78 95% CI 0.66, 0.92).

**Conclusions:**

Overall, we found no evidence of increased risks of cancer-specific mortality in breast, colorectal or prostate cancer patients who used ACEI or ARBs after diagnosis. These results provide some reassurance that these medications are safe in patients diagnosed with these cancers.

## Background

Angiotensin-converting enzyme inhibitors (ACEIs) and angiotensin II receptor blockers (ARBs) are commonly prescribed to the large and growing number of individuals with cancer (for example, currently more than two million in the UK [1] and 13 million in the US [2]) often to treat hypertension which affects around 40% of cancer patients [3]. However, the possible effect of ACEIs and ARBs on cancer is subject to much debate. Concerns were first raised in 2003 when the Candesartan in Heart Failure Assessment of Reduction in Mortality and Morbidity (CHARM) trial [4] unexpectedly reported a significant 40% increase in fatal cancer in patients randomized to candesartan (an ARB) compared with placebo (relative risk (RR) = 1.42 95% CI 1.02, 1.98; *P* = 0.04). In part conducted because of this finding, a 2010 meta-analysis of randomized trials observed increased cancer risk with ARBs [5]. Preclinical studies also suggested a biological rationale for an increase in cancer risk in ARB users [6,7]. A later meta-analysis observed no association for ARBs but observed a small increased risk of cancer in combination ARB and ACEI users [8]. These findings for cancer risk were further investigated in large population-based observational studies [9-12] which, although generally negative, observed small increases in cancer risk, for instance, for breast and prostate cancer in ARB users [12] and lung cancer in ACEI users [11].

These results, and particularly the increases in fatal cancer observed in the 2003 ARB trial [4], raise questions about the safety of ACEI and ARB use in cancer patients. Despite these findings, there have not been any studies which have investigated ARB use separately after cancer diagnosis and cancer progression in prostate or colorectal cancer patients and only two studies have investigated the specific association between ARB use and cancer progression in breast cancer patients [13,14]. ACEIs and cancer progression has also received little attention with only two studies of ACEI use in colorectal cancer patients [15,16] and one in prostate cancer patients [17]. Five studies have investigated ACEI use in breast cancer patients and cancer progression but have reached conflicting results [13,14,18-20]. Further investigation of ACEIs and ARBs and cancer progression is, therefore, warranted.

This study investigated whether ACEI or ARB use after diagnosis of breast, colorectal or prostate cancer was associated with increased cancer-specific, or all cause, mortality in large population-based cohorts of cancer patients.

## Methods

### Study design

Cohort studies were conducted utilizing linkages between the English National Cancer Data Repository (NCDR), the UK Clinical Practice Research Datalink (CPRD) and the Office of National Statistics (ONS) death registrations. The NCDR data include date and site of primary cancer diagnosis and clinical data, such as stage and treatment. The CPRD is the world’s largest database of longitudinal patient records comprising around 8% of the UK population and includes demographic information, clinical diagnoses and prescription data which is of documented high quality [21,22]. Ethical approval for all observational research using CPRD data has been obtained from a multicenter research ethics committee [23]. Linkages between the datasets were conducted using a deterministic algorithm based upon National Health Service (NHS) number, gender, date of birth and postcode. Patients were included in the cohorts if they had a CPRD diagnosis code for breast (women only), colorectal or prostate cancer which was confirmed by NCDR cancer diagnosis (based upon International Classification of Diseases (ICD) codes of C50 for breast cancer, C61 for prostate cancer, and C18, C19 and C20 for colorectal cancer) from 1998 to 2006. Cancer patients with previous NCDR cancer diagnosis, apart from *in situ* neoplasms and non-melanoma skin cancers, were excluded. Cancer patients were also excluded if the date of cancer diagnosis preceded CPRD research quality records. Date and cause of death up to 2011 were taken from ONS. Analysis was restricted to individuals with available ONS mortality data from cancer diagnosis.

### ACEI\ARB identification

ACEIs and ARBs were defined as all agents within the two drug classes according to the British National Formulary [24] (BNF, chapters 2.5.5.1 and 2.5.5.2, respectively). ACEI and ARB prescriptions within the cohorts from CPRD prescribing data were counted and converted to daily defined doses (DDD) on the basis of the quantity and strength (as defined by the World Health Organization [25]). A quantity of 28 tablets was assumed for approximately 2% of prescriptions where quantity was missing or inconsistent. Medication usage was ascertained in the exposure period described later.

### Potential confounders

Data available from the NCDR included stage, histological grade, Gleason score (for prostate cancer), surgery, chemotherapy and radiotherapy in the six months after diagnosis. Gleason score was converted to grade to increase completeness [26]. General practitioner (GP) prescribing data were used to determine hormone therapy in the first six months after cancer diagnosis including androgen therapy for prostate cancer (BNF chapter 8.3.4.2, including gonadorelin analogues and anti-androgens) and tamoxifen and aromatase inhibitors for breast cancer (BNF chapter 8.3.4.1). Breast and prostate cancer patients were excluded if hormone therapy preceded cancer diagnosis by eight weeks. In breast cancer patients, hormone replacement therapy (HRT) for estrogen and progestogens (BNF chapters 6.4.1. and 6.4.2.) was determined prior to diagnosis. Low dose aspirin and statin use were taken from GP prescription records. Smoking, alcohol intake and body mass index (BMI) were determined from the closest GP record prior to cancer diagnosis (records older than ten years were ignored). Comorbidities were determined from GP diagnosis codes on the basis of diagnoses contributing to a recent adaptation of the Charlson comorbidity index for GPRD [27].

### Data analysis

The cancer cohorts were initially analyzed using a time matched nested case–control approach, a common approach, for example [28], which accounts for immortal time bias [29] without requiring complicated statistical techniques [30] with minimal loss of precision [31], and a time varying covariate approach, described later. Breast cancer cases were members who had died due to breast cancer (with an ICD code of C50 as the underlying cause of death) and these were matched on age (in five year intervals) and year of cancer diagnosis to five risk-set controls who lived at least as long after their cancer diagnosis. Corresponding analyses were conducted for colorectal cancer cases (ICD codes of C18, C19, C20, C21 or C26 as their underlying cause of death) who were matched to risk-set controls on gender, site (colon or rectal), age (in five year intervals) and year of cancer diagnosis (in two year intervals) and prostate cancer cases (with ICD codes of C61 as their underlying cause of death) who were matched to risk set controls on age (in five year intervals) and year of cancer diagnosis.

The exposure period (for identification of post-diagnostic medication usage) in cases was the period from cancer diagnosis until six months prior to cancer-specific death. The exposure period in the controls was fixed to be the same duration as that of their matched cases and began at the date of cancer diagnosis in the control. The exposure period did not include prescriptions in the six month period prior to death as these may reflect end of life treatment or increased exposure to healthcare professionals (sensitivity analyses investigated increasing this to 12 months). Analyses were restricted to individuals with at least one year of follow-up after cancer diagnosis.

Conditional logistic regression was used to estimate the odds of death from cancer in cancer patients prescribed one or more ACEIs in the exposure period compared to those with none and corresponding odds ratios (ORs) and 95% confidence intervals (95% CIs) were determined before and after adjustment for potential confounders. Similar analyses were conducted by duration of medication usage (investigating individuals prescribed over 365 DDDs of ACEIs in the exposure period equivalent to one year of usage). These analyses were repeated for ARBs. An analysis of all-cause mortality was also conducted in which cancer patients who died from any-cause were matched to risk-set controls (using the same matching criteria as in the cancer-specific analyses) and conditional logistic regression models were applied as described previously. A secondary analysis was conducted to investigate pre-diagnostic ACEI/ARB use. Two sensitivity analyses were conducted which restricted the non-user group to patients with more similar indications. In one analysis, the cancer cohorts were first restricted to individuals using any antihypertensive medications in the year prior to cancer diagnosis (including diuretics, vasodilator antihypertensive drugs, centrally acting antihypertensive drugs, alpha-adrenoceptor blocking drugs, beta-blockers, ACEIs, ARBs, renin inhibitors, and calcium channel blockers) and the analysis was conducted as previously. In the other sensitivity analysis, the full cohort was used as in the main analysis but ORs were calculated comparing ACEI/ARB users in the exposure period not to ACEI/ARB non-users in the exposure period (as in the main analysis) but to ACEI/ARB non-users who had used at least one other antihypertensive medication in the exposure period. Sensitivity analyses were also conducted additionally adjusting for other antihypertensive use and in breast and prostate cancer patients, adjusting for hormone therapy at any time after diagnosis (not just in the first six months after diagnosis as in the main analysis). A sensitivity analyses was conducted in prostate cancer patients adjusting for Gleason score and in breast cancer patients restricted to cancer registries with high rates of available stage (overall, over 85% complete). Stratified analyses were conducted by stage (for colorectal and breast cancer), sex (for colorectal) and site (for colorectal cancer). Stratified analyses were conducted after re-matching cases to controls within strata. Interaction tests were used to compare associations between strata [32].

Additionally, the cancer cohort was analyzed, without conversion to case–control data, applying survival analysis to investigate ACEI/ARB exposure as a time varying covariate (individuals were considered non-users prior to use and users after a lag of six months after first use, to mimic the case–control analysis) [29]. A similar dose response analysis was conducted with individuals considered non-users prior to six months after first use, a short term user between six months after first use and six months after 365 DDDs and a longer term user after this time. A separate analysis was conducted additionally adjusting for competing risk of deaths using Fine and Gray’s proportional subhazards model (not shown, as results similar) [33].

A pre-study power calculation based upon 15% ACEI use and predicted numbers suggested the study would have over 80% power to detect an OR of 1.20 for cancer-specific mortality in patients receiving ACEIs in each of the cancer cohorts (breast, colorectal, prostate). More accurately, based upon the final numbers (shown in Table 1) and ACEI and ARB use (shown in Table 2), the study had approximately 80% power to detect, at the 5% significance level, an OR for cancer-specific mortality in ACEI users of 1.25 and for ARB users of 1.35 in each of the cancer cohorts. Statistical analyses were conducted in STATA 11 (StataCorp, College Station, Texas).

**Table 1:** Characteristics of cancer patients who die from cancer (cases) compared with controls, by cancer site

	**Breast cancer**			**Colorectal cancer**			**Prostate cancer**		
	**Cases**	**Controls**	***P***	**Cases**	**Controls**	***P***	**Cases**	**Controls**	***P***
	**number (%)**	**number (%)**		**number (%)**	**number (%)**		**number (%)**	**number (%)**	
Year of diagnosis									
1998 to 2000	500 (35)	2,466 (35)	N.A.	379 (25)	1,842 (25)	N.A.	396 (33)	1,941 (33)	N.A.
2001 to 2003	518 (36)	2,570 (36)		564 (37)	2,721 (37)		461 (39)	2,281 (39)	
2003 to 2006	417 (29)	2,070 (29)		568 (38)	2,728 (37)		327 (28)	1,627 (28)	
Age at diagnosis (years)									
<50	339 (24)	1,667 (23)	N.A.	90 (6)	330 (5)	N.A.	6 (1)	20 (0)	N.A.
50 to 59	286 (20)	1,430 (20)		226 (15)	1,117 (15)		71 (6)	351(6)	
60 to 69	273 (19)	1,365 (19)		401 (27)	2,001 (27)		280 (24)	1,400 (24)	
70 to 79	318 (22)	1,588 (22)		496 (33)	2,472 (34)		532 (45)	2,660 (45)	
≥ 80	219 (15)	1,056 (15)		298 (20)	1,371 (19)		295 (25)	1,418 (24)	
Male	0 (0)	0 (0)	N.A.	871 (58)	4,231 (58)	N.A.	1,184(100)	5,849 (100)	N.A.
Exposure period (years):									
Mean (sd)	3.9 (2.3)	3.9 (2.3)	N.A.	2.8 (1.6)	2.9 (1.7)	N.A.	3.8 (2.2)	3.7 (2.2)	N.A.
Range	1.0 to 12.8	1.0 to 12.8	N.A.	1.0 to 10.5	1.0 to 10.5	N.A.	1.0 to 11.9	1.0 to 11.9	
Stage:1	72 (11)	1,471 (43)	<0.001	61 (6)	1,044 (18)	<0.001			
2	402 (61)	1,676 (49)		275 (25)	2,496 (44)				
3	116 (18)	220 (6)		558 (51)	1,981 (35)				
4	64 (10)	40 (1)		203 (19)	195 (3)				
Missing	781	3,699		414	1,575				
Grade:Well	54 (6)	751 (19)		81 (7)	562 (9)	<0.001	34 (4)	538 (13)	<0.001
Moderate	377 (41)	1,900 (49)		889 (75)	4,894 (78)		217 (28)	2,067 (48)	
Poor	492 (53)	1,217 (31)		208 (17)	818 (13)		516 (67)	1,683 (39)	
Missing	512	3,280		333	1017		417	1561	
Treatment within six months of diagnosis									
Chemotherapy	573 (40)	1,611 (23)	<0.001	651 (43)	1,916 (26)	<0.001	49 (4)	137 (2)	<0.001
Radiotherapy	700 (49)	3,355 (47)	0.22	302 (20)	1,066 (15)	<0.001	246 (21)	1,276 (22)	0.48
Surgery	1,087 (76)	5,982 (84)	<0.001	1,214 (80)	65,15 (89)	<0.001			
Tamoxifen therapy	709 (49)	4,610 (65)	<0.001						
Androgen therapy							976 (82)	3,450 (59)	<0.001
Radical prostatectomy^a^							20 (2)	331 (8)	<0.001
Smoking prior to diagnosis			0.03			0.01			<0.001
Non-smoker	710 (60)	3,827 (64)		640 (51)	3,138 (52)		453 (47)	2,541 (52)	
Ex-smoker	226 (19)	1,072 (18)		365 (29)	1,885 (31)		331 (34)	1,660 (34)	
Current smoker	249 (21)	1,094 (18)		249 (20)	983 (16)		185 (19)	674 (14)	
Missing	250	1,113		257	1285		215	974	
BMI prior to diagnosis			0.01			0.66			0.05
Mean (sd)	26.6 (5.5)	26.2 (5.1)		26.5 (4.7)	26.6 (4.8)		26.4 (4.0)	26.1 (3.7)	
Missing	386	1,633		412	1,810		302	1,362	
Alcohol prior to diagnosis			0.58			0.47			0.37
Alcohol consumer	831 (80)	4,333 (81)		950 (87)	4,585 (86)		791 (89)	4,047 (90)	
Missing	401	1,779		420	1,977		298	1366	
Comorbidity (prior to diagnosis or during follow-up time)									
Cerebrovascular	89 (6)	383 (5)	0.53	117 (8)	493 (7)	0.19	124 (10)	509 (9)	0.07
C.P.D.	264 (18)	1,247 (18)	0.60	236 (16)	1295 (18)	0.06	229 (19)	1,110 (19)	0.80
C.H.D.	60 (4)	249 (4)	0.68	91 (6)	367 (5)	0.13	108 (9)	395 (7)	0.01
Diabetes	126 (9)	468 (7)	0.02	192 (13)	866 (12)	0.21	141 (12)	619 (11)	0.17
Myocardial infarction	26 (2)	148 (2)	0.74	104 (7)	454 (6)	0.32	130 (11)	522 (9)	0.03
Peptic ulcer disease	39 (3)	184 (3)	0.79	111 (7)	477 (7)	0.19	77 (7)	440 (8)	0.18
P.V.D.	51 (4)	165 (2)	0.003	64 (4)	331 (5)	0.70	113 (10)	396 (7)	0.001
Renal disease	78 (5)	382 (5)	0.92	107 (7)	478 (7)	0.45	138 (12)	509 (9)	0.001
Rheumatological	76 (5)	330 (5)	0.35	55 (4)	272 (4)	0.82	37 (3)	287 (5)	0.01

^a^Excludes cancer registries without available data. C.P.D., chronic pulmonary disease; C.H.D., congestive heart disease; N.A., not applicable; P.V.D., peripheral vascular disease; sd, standard deviation.

**Table 2:** Association between post-diagnostic exposure to ACEIs and ARBS and cancer specific death, by cancer site

**Post-diagnostic medication usage**	**All cancer patients**						**Cancer patients with available stage/grade**^**c**^			
	**Cancer specific deaths number (%)**	**Controls number (%)**	**Unadjusted OR (95% CI)**	***P *** **(trend**^**a**^**)**	**Adjusted**^**b **^**OR (95% CI)**	***P *** **(trend**^**a**^**)**	**Unadjusted OR (95% CI)**	***P *** **(trend**^**a**^**)**	**Fully adjusted OR (95% CI)**^**d**^	***P *** **(trend**^**a**^**)**
			**Breast cancer**							
**ACEI**										
0 (non-user)	1,192 (83.1)	5,938 (83.6)	1.00 (ref. cat.)		1.00 (ref. cat.)		1.00 (ref. cat.)		1.00 (ref. cat.)	
≥1 DDD (user)	243 (16.9)	1,168 (16.4)	1.04 (0.88, 1.22)	0.65	1.06 (0.89, 1.27)	0.52	0.96 (0.76, 1.21)	0.72	0.83 (0.63, 1.09)	0.18
				(0.50)		(0.32)		(0.88)		(0.47)
1to 365 DDDs	92 (6.4)	469 (6.6)	0.97 (0.77, 1.23)	0.80	0.96 (0.75, 1.24)	0.78	0.88 (0.61, 1.27)	0.49	0.65 (0.43, 0.99)	0.04
≥365 DDDs	151 (10.5)	699 (9.8)	1.08 (0.89, 1.32)	0.42	1.14 (0.92, 1.41)	0.25	1.01 (0.76, 1.33)	0.96	0.96 (0.69, 1.34)	0.82
**ARB**										
0 (non-user)	1,333 (92.9)	6,602 (92.9)	1.00 (ref. cat.)		1.00 (ref. cat.)		1.00 (ref. cat.)		1.00 (ref. cat.)	
≥1 DDD (user)	102 (7.1)	504 (7.1)	1.00 (0.80, 1.26)	0.98	1.06 (0.84, 1.35)	0.62	1.03 (0.84, 1.25)	0.80	0.94 (0.65, 1.37)	0.75
				(0.99)		(0.57)		(0.69)		(0.85)
1 to 365 DDDs	33 (2.3)	160 (2.3)	1.02 (0.70, 1.51)	0.87	1.01 (0.68, 1.51)	0.96	1.04 (0.58, 1.87)	0.90	0.84 (0.44, 1.61)	0.59
≥365 DDDs	69 (4.8)	344 (4.8)	0.99 (0.75, 1.30)	0.95	1.09 (0.82, 1.45)	0.55	1.08 (0.73, 1.60)	0.70	0.99 (0.64, 1.52)	0.95
			**Colorectal cancer**							
**ACEI**										
0 (non-user)	1,231 (81.5)	5,660 (77.6)	1.00 (ref. cat.)		1.00 (ref. cat.)		1.00 (ref. cat.)		1.00 (ref. cat.)	
≥1 DDD (user)	280 (18.5)	1,631 (22.4)	0.79 (0.69, 0.92)	0.002	0.78 (0.66, 0.92)	0.003	0.75 (0.63, 0.89)	0.001	0.76 (0.62, 0.93)	0.01
				(0.01)		(0.02)		(0.005)		(0.02)
1 to 365 DDDs	113 (7.5)	729 (10.0)	0.72 (0.59, 0.89)	0.002	0.71 (0.57, 0.89)	0.03	0.69 (0.54, 0.90)	0.005	0.71 (0.53, 0.94)	0.02
≥365 DDDs	167 (11.1)	902 (12.4)	0.85 (0.71, 1.02)	0.09	0.84 (0.68, 1.03)	0.10	0.80 (0.64, 0.98)	0.03	0.80 (0.63, 1.02)	0.07
**ARB**										
0 (non-user)	1,428 (94.5)	6,811 (93.4)	1.00 (ref. cat.)		1.00 (ref. cat.)		1.00 (ref. cat.)		1.00 (ref. cat.)	
≥1 DDD (user)	83 (5.5)	480 (6.6)	0.84 (0.66, 1.07)	0.15	0.82 (0.64, 1.07)	0.14	0.91 (0.76, 1.08)	0.29	0.80 (0.59, 1.09)	0.16
				(0.41)		(0.94)		(0.26)		(0.66)
1 to 365 DDDs	29 (1.9)	221 (3.0)	0.64 (0.43, 0.95)	0.03	0.63 (0.42, 0.94)	0.03	0.51 (0.31, 0.83)	0.01	0.61 (0.37, 1.03)	0.06
≥365 DDDs	54 (3.6)	259 (3.6)	1.00 (0.74, 1.36)	0.98	1.00 (0.72, 1.37)	0.98	0.98 (0.69, 1.38)	0.89	0.93 (0.64, 1.36)	0.72
			**Prostate cancer**							
**ACEI**										
0 (non-user)	848 (71.6)	4,145 (70.9)	1.00 (ref. cat.)		1.00 (ref. cat.)		1.00 (ref. cat.)		1.00 (ref. cat.)	
≥1 DDD (user)	336 (28.4)	1,704 (29.1)	0.96 (0.83, 1.10)	0.55	0.78 (0.66, 0.92)	0.003	0.98 (0.82, 1.17)	0.80	0.82 (0.67, 1.02)	0.07
				(0.47)		(0.003)		(0.85)		(0.11)
1 to 365 DDDs	118 (10.0)	572 (9.8)	1.00 (0.81, 1.24)	0.99	0.83 (0.66, 1.04)	0.11	0.96 (0.74, 1.26)	0.79	0.80 (0.59, 1.07)	0.14
≥365 DDDs	218 (18.4)	1,132 (19.4)	0.94 (0.79, 1.11)	0.44	0.75 (0.62, 0.91)	0.003	0.98 (0.80, 1.21)	0.88	0.84 (0.66, 1.07)	0.16
**ARB**										
0 (non-user)	1,103 (93.2)	5,384 (92.1)	1.00 (ref. cat.)		1.00 (ref. cat.)		1.00 (ref. cat.)		1.00 (ref. cat.)	
≥1 DDD (user)	81 (6.8)	465 (8.0)	0.86 (0.67, 1.10)	0.22	0.79 (0.61,1.03)	0.08	0.93 (0.70, 1.23)	0.62	0.82 (0.61, 1.11)	0.21
				(0.16)		(0.06)		(0.75)		(0.26)
1 to 365 DDDs	30 (2.5)	153 (2.6)	0.97 (0.65, 1.44)	0.88	0.88 (0.58, 1.33)	0.55	0.84 (0.52, 1.36)	0.48	0.77 (0.46, 1.29)	0.33
≥365 DDDs	51 (4.3)	312 (5.3)	0.80 (0.59, 1.09)	0.15	0.74 (0.54, 1.02)	0.07	0.98 (0.70, 1.36)	0.90	0.84 (0.59, 1.21)	0.36

^a^Tests for trend from conditional logistic regression model based upon categories of DDDs (0, 1 to 365, ≥365 DDDs coded as 0, 1, 2, respectively). ^b^All models include surgery (within six months of diagnosis), chemotherapy (within six months), radiotherapy (within six months), low dose aspirin (during exposure period), statins (during exposure period), comorbidities (pre-diagnosis or during exposure period, including myocardial infarction, cerebrovascular disease, congestive heart disease, chronic pulmonary disease, peripheral vascular disease, renal disease, peptic ulcer disease and diabetes), and smoking (pre-diagnosis, with missing included as a category). The breast cancer model additionally includes tamoxifen (within six months), aromatase inhibitors (within six months), hormone replacement therapy (pre-diagnosis). The prostate cancer model additionally includes androgen therapy (within six months). ^c^Analysis includes 1,093 cases and 5,231 controls in colorectal cancer patients with available stage, 648 cases and 3,193 controls in breast cancer patients with available stage and 766 cases and 3,777 controls in prostate cancer patients with available grade. ^d^Additionally adjusted for stage in colorectal cancer patients and breast cancer patients and adjusted for grade in prostate cancer patients. ACEIs, angiotensin-converting enzyme inhibitors; ARBs, angiotensin II receptor blockers; CI, confidence interval; DDDs, daily defined doses; OR, odds ratio.

## Results

### Patient cohorts

**Figure 1:**
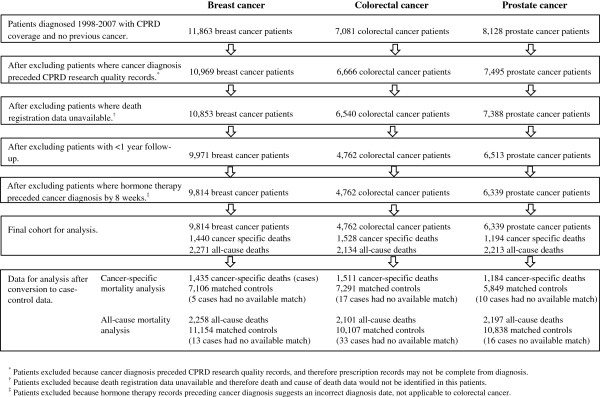
Flow chart of selection of patients for inclusion in analysis, by cancer site.

Figure 1 shows the selection of the final cohorts. The average follow-up (in those not dying) was six years (range one to thirteen years) in each of the three cohorts. The crude rate of cancer-specific death was 28.0 per 1,000 person years in the breast cancer cohort (based upon 1,440 breast cancer-specific deaths in 51,507 person years), 78.5 per 1,000 person years in the colorectal cancer cohort (based upon 1,528 colorectal cancer-specific deaths in 19,462 person years), and 41.2 per 1,000 person years in the prostate cancer cohort (based upon 1,194 prostate cancer-specific deaths in 28,970 person years). These cohorts were converted to case–control data for the cancer-specific mortality analysis with 1,435 breast, 1,511 colorectal and 1,184 prostate cases (cancer-specific deaths) and 7,106 breast, 7,291 colorectal and 5,849 prostate risk-set controls.

### Patient characteristics

Table 1 shows characteristics of cancer-specific deaths (cases) and controls. The average time to death and, hence, the end of the exposure period was 3.9, 2.8 and 3.8 years for breast, colorectal and prostate cancer, respectively. Cancer-specific deaths (cases) had higher stage, higher histological grade, more chemotherapy and less surgery. A higher proportion of cases had radiotherapy for colorectal but not breast or prostate cancer. Hormone therapy was less frequent in breast cancer cases but more frequent in prostate cancer cases. Smoking rates were slightly higher in cases. Rates of comorbidities, alcohol consumption and BMI levels prior to diagnosis were generally similar between cases and controls (Table 1).

### ACEI/ARB use and mortality in breast cancer patients

In breast cancer patients (Table 2), the ACEI prescriptions after diagnosis were similar in those dying from cancer (cases) compared with controls (16.9% versus 16.4%, respectively; OR = 1.04, 95% CI 0.88, 1.22). Adjustment for potential confounders did not alter this OR markedly (OR = 1.06, 95% CI 0.89, 1.27). Similarly, no associations were observed between cancer-specific mortality and ARB use (adjusted OR = 1.06, 95% CI 0.84, 1.35). These findings were little altered after adjustment for stage and were fairly consistent across sensitivity analyses (shown in Table 3). No associations were observed with pre-diagnostic use (Table 3). Finally, analyses of post-diagnostic ACEI (adjusted OR = 1.04, 95% CI 0.92, 1.19) and ARB (adjusted OR = 0.96, 95% CI 0.81, 1.14) use and all-cause mortality showed little evidence of associations (Table 4).

**Table 3:** Sensitivity analyses for association between ACEIs and ARBS and cancer specific mortality in breast cancer patients

	**Cancer specific deaths number**	**Controls number**	**OR (95% CI)**	***P***	**OR (95% CI)**	***P***
			**ACEI user versus non user**		**ARB user versus non user**	
**Breast cancer**						
Main analysis: diagnosis to six months prior to death^a^	648	3,193	0.83 (0.63, 1.09)	0.18	0.94 (0.65, 1.37)	0.75
Diagnosis to 1 year prior to death^b^	583	2,875	0.87 (0.65, 1.16)	0.33	0.87 (0.58, 1.30)	0.49
Restricted to users of any antihypertensive medication^c^ prior to cancer diagnosis^d^	212	994	0.87 (0.60, 1.25)	0.44	1.36 (0.85, 2.16)	0.20
Comparison group restricted to users of any antihypertensive in exposure period^c^	361	1,624	0.79 (0.59, 1.05)	0.10	0.92 (0.63, 1.34)	0.67
Additionally adjusting for other antihypertensives^e^	648	3,193	0.79 (0.60, 1.06)	0.12	0.90 (0.61, 1.33)	0.60
Additionally adjusting for hormone therapy any time after diagnosis^f^	648	3,193	0.80 (0.60, 1.05)	0.11	0.98 (0.66, 1.44)	0.91
≥730 DDDs versus 0 DDDs (non-user)	648	3,193	1.06 (0.74, 1.52)	0.75	0.99 (0.64, 1.52)	0.95
Restricted to cancer registries with stage data available for over 85% of patients	487	1,911	0.84 (0.59, 1.18)	0.31	1.33 (0.81, 2.17)	0.25
Stage 1 and 2^g^	469	2,313	0.95 (0.68, 1.32)	0.75	1.14 (0.73, 1.81)	0.56
Stage 3 and 4^g^	161	531	0.83 (0.46, 1.51)	0.54	2.37 (1.05, 5.35)	0.04
Pre-diagnostic use^h^	695	3,413	0.86 (0.61, 1.20)	0.37	1.06 (0.65, 1.72)	0.82
Time varying covariate analysis^i^	656	4,164				
≥1 DDDs versus 0 DDDs (non-user)			0.97 (0.77, 1.21)	0.76	1.20 (0.89, 1.62)	0.23
1to 365 DDDs versus 0 DDDs (non-user)			0.90 (0.65, 1.25)	0.52	0.94 (0.55, 1.60)	0.81
≥365 DDDs versus 0 DDDs (non-user)			1.01 (0.77, 1.32)	0.93	1.35 (0.96, 1.91)	0.09

^a^Except where otherwise stated analysis investigates medications from diagnosis to six months prior to death/index date and models include surgery (within six months of diagnosis), chemotherapy (within six months), radiotherapy (within six months), low dose aspirin (during exposure period), statins (during exposure period), comorbidities (pre-diagnosis or during exposure period, including myocardial infarction, cerebrovascular disease, congestive heart disease, chronic pulmonary disease, peripheral vascular disease, renal disease, peptic ulcer disease and diabetes), and smoking (pre-diagnosis, with missing included as a category), tamoxifen (within six months), aromatase inhibitors (within six months), hormone replacement therapy (pre-diagnosis) and stage. ^b^Restricted to individuals surviving more than 1.5 years. ^c^Antihypertensive medications include beta-blockers, diuretics, vasodilator antihypertensive drugs, centrally acting antihypertensive drugs, alpha-adrenoceptor blocking drugs, ACEIs, ARBs, renin inhibitors, and calcium channel blockers. ^d^Restricted to individuals with >1 year of records prior to cancer diagnosis and pre-diagnostic use considered antihypertensive use in that year, excluding deaths in the year after cancer diagnosis. ^e^Models include all variables in ^a^and additionally include calcium channel blockers, diuretics and beta-blockers. ^f^Models include all variables in ^a^but tamoxifen and aromatase inhibitors were determined at any time after diagnosis in the exposure period. ^g^Stratified analyses were conducted after re-matching controls to cases within strata; due to non-availability of matches overall numbers in subgroups may not be identical to numbers presented in Table 1. ^h^Restricted to individuals with >1 year of records prior to cancer diagnosis and pre-diagnostic use considered ACEI/ARB use in that year, not excluding deaths in the year after cancer diagnosis. ^i^Reported estimates are adjusted hazard ratios and 95% CIs, model includes age at diagnosis, year of diagnosis, surgery (within six months of diagnosis), chemotherapy (within six months), radiotherapy (within six months), statins (during exposure period, as a time varying covariate), comorbidities (pre-diagnosis including, myocardial infarction, cerebrovascular disease, congestive heart disease, chronic pulmonary disease, peripheral vascular disease, renal disease, peptic ulcer disease and diabetes) and stage. ACEIs, angiotensin-converting enzyme inhibitors; ARBs, angiotensin II receptor blockers; CI, confidence interval; DDDs, daily defined doses; OR, odds ratio.

**Table 4:** Association between post-diagnostic exposure to ACEIs and ARBS and all-cause mortality, by cancer site

**Post-diagnostic medication usage**	**All cancer patients**						**Cancer patients with available stage/grade**^**b**^			
	**All-cause deaths number (%)**	**Controls number (%)**	**Unadjusted OR (95% CI)**	***P *** **(trend**^**d**^**)**	**Adjusted**^**a **^**OR (95% CI)**	***P *** **(trend**^**d**^**)**	**Unadjusted OR (95% CI)**	***P *** **(trend**^**d**^**)**	**Fully adjusted OR (95% CI)**^**c**^	***P *** **(trend**^**d**^**)**
		**Breast cancer**								
**ACEI**										
0 (non-user)	1,749 (77.5)	8,948 (80.2)	1.00 (ref. cat.)		1.00 (ref. cat.)		1.00 (ref. cat.)		1.00 (ref. cat.)	
≥1 DDD (user)	509 (22.5)	2,206 (19.8)	1.19 (1.06, 1.34)	0.003	1.04 (0.92, 1.19)	0.51	1.19 (1.00, 1.41)	0.05	0.93 (0.76, 1.14)	0.49
				(0.01)		(0.60)		(0.09)		(0.37)
1 to 365 DDDs	193 (8.6)	821 (8.6)	1.20 (1.02, 1.42)	0.03	1.06 (0.89, 1.27)	0.52	1.26 (0.97, 1.62)	0.08	1.00 (0.76, 1.34)	0.97
≥365 DDDs	316 (14.0)	1,385 (12.4)	1.18 (1.03, 1.36)	0.02	1.03 (0.89, 1.20)	0.68	1.15 (0.94, 1.41)	0.17	0.89 (0.71, 1.13)	0.34
**ARB**										
0 (non-user)	2,057 (91.1)	10,161 (91.1)	1.00 (ref. cat.)		1.00 (ref. cat.)		1.00 (ref. cat.)		1.00 (ref. cat.)	
≥1 DDD (user)	201 (8.9)	993 (8.9)	1.00 (0.85, 1.18)	0.99	0.96 (0.81, 1.14)	0.62	0.91 (0.71, 1.16)	0.45	0.79 (0.60 ,1.03)	0.08
				(0.85)		(0.80)		(0.69)		(0.18)
1 to 365 DDDs	64 (2.8)	338 (3.0)	0.93 (0.70, 1.22)	0.59	0.88 (0.66, 1.17)	0.37	0.73 (0.47, 1.15)	0.18	0.62 (0.39, 1.00)	0.05
≥365 DDDs	137 (6.1)	655 (5.9)	1.04 (0.85, 1.26)	0.71	1.00 (0.82, 1.23)	0.99	1.00 (0.75, 1.33)	0.99	0.87 (0.64, 1.19)	0.38
		**Colorectal cancer**								
**ACEI**										
0 (non-user)	1,596 (76.0)	7,578 (75.0)	1.00 (ref. cat.)		1.00 (ref. cat.)		1.00 (ref. cat.)		1.00 (ref. cat.)	
≥1 DDD (user)	505 (24.0)	2,529 (25.0)	0.96 (0.85, 1.07)	0.44	0.90 (0.79, 1.02)	0.10	0.94 (0.82, 1.07)	0.36	0.89 (0.76, 1.03)	0.12
				(0.31)		(0.05)		(0.38)		(0.10)
1 to 365 DDDs	206 (9.8)	985 (9.8)	1.00 (0.86, 1.18)	0.95	0.95 (0.80, 1.13)	0.58	0.94 (0.78, 1.14)	0.55	0.92 (0.75, 1.13)	0.41
≥365 DDDs	299 (14.2)	1,544 (15.3)	0.92 (0.80, 1.06)	0.27	0.85 (0.73, 1.00)	0.05	0.94 (0.80, 1.10)	0.43	0.86 (0.72, 1.04)	0.11
**ARB**										
0 (non-user)	1,939 (92.3)	9,294 (92.0)	1.00 (ref. cat.)		1.00 (ref. cat.)		1.00 (ref. cat.)		1.00 (ref. cat.)	
≥1 DDD (user)	162 (7.8)	813 (8.0)	0.97 (0.82, 1.16)	0.78	0.94 (0.78, 1.13)	0.50	1.01 (0.82, 1.23)	0.95	1.02 (0.82, 1.26)	0.88
				(0.88)		(0.86)		(0.66)		(0.72)
1 to 365 DDDs	59 (2.8)	344 (3.4)	0.84 (0.64, 1.12)	0.24	0.80 (0.60, 1.06)	0.12	0.85 (0.61, 1.19)	0.35	0.92 (0.65, 1.31)	0.66
≥365 DDDs	103 (4.9)	469 (4.6)	1.07 (0.86, 1.34)	0.54	1.05 (0.83, 1.32)	0.70	1.11 (0.86, 1.41)	0.42	1.07 (0.83, 1.39)	0.60
		**Prostate cancer**								
**ACEI**										
0 (non-user)	1,463 (66.6)	7,512 (69.3)	1.00 (ref. cat.)		1.00 (ref. cat.)		1.00 (ref. cat.)		1.00 (ref. cat.)	
≥1 DDD (user)	734 (33.4)	3,326 (30.7)	1.14 (1.03, 1.26)	0.01	0.91 (0.82, 1.03)	0.13	1.09 (0.97, 1.23)	0.15	0.88 (0.76, 1.00)	0.06
				(0.04)		(0.05)		(0.13)		(0.07)
1 to 365 DDDs	258 (11.7)	1,098 (10.1)	1.20 (1.04, 1.40)	0.01	1.00 (0.85, 1.17)	0.96	1.06 (0.89, 1.27)	0.53	0.88 (0.73, 1.07)	0.20
≥365 DDDs	476 (21.7)	2,228 (20.6)	1.11 (0.98, 1.24)	0.09	0.87 (0.76, 0.99)	0.04	1.11 (0.97, 1.27)	0.14	0.87 (0.75, 1.02)	0.09
**ARB**										
0 (non-user)	2,002 (91.1)	9,873 (91.1)	1.00 (ref. cat.)		1.00 (ref. cat.)		1.00 (ref. cat.)		1.00 (ref. cat.)	
≥1 DDD (user)	195 (8.9)	965 (8.9)	1.00 (0.85, 1.18)	0.98	0.85 (0.72, 1.01)	0.06	1.08 (0.89, 1.30)	0.45	0.92 (0.75, 1.12)	0.39
				(0.99)		(0.07)		(0.33)		(0.56)
1 to 365 DDDs	61 (2.8)	297 (2.7)	1.02 (0.77, 1.35)	0.89	0.86 (0.64, 1.15)	0.32	0.95 (0.68, 1.33)	0.77	0.81 (0.57, 1.14)	0.26
≥365 DDDs	134 (6.1)	668 (6.2)	0.99 (0.82, 1.21)	0.95	0.84 (0.69, 1.03)	0.10	1.14 (0.91, 1.43)	0.26	0.97 (0.76, 1.23)	0.81

^a^All models include surgery (within six months of diagnosis), chemotherapy (within six months), radiotherapy (within six months), low dose aspirin (during exposure period), statins (during exposure period), comorbidities (pre-diagnosis or during exposure period, including myocardial infarction, cerebrovascular disease, congestive heart disease, chronic pulmonary disease, peripheral vascular disease, renal disease, peptic ulcer disease and diabetes), and smoking (pre-diagnosis, with missing included as a category). The breast cancer model additionally includes tamoxifen (within six months), aromatase inhibitors (within six months), hormone replacement therapy (pre-diagnosis). The prostate cancer model additionally includes androgen therapy (within six months). ^b^Analysis includes 1,558 cases and 7,406 controls in colorectal cancer patients with available stage, 1,008 cases and 4,900 controls in breast cancer patients with available stage and 1,520 cases and 7,473 controls in prostate cancer patients with available grade. ^c^Additionally adjusted for stage in colorectal cancer patients and breast cancer patients and adjusted for grade in prostate cancer patient. ^d^Tests for trend from conditional logistic regression model based upon categories of DDDs (0, 1 to365, ≥365 DDDs coded as 0, 1, 2 respectively). ACEIs, angiotensin-converting enzyme inhibitors; ARBs, angiotensin II receptor blockers; CI, confidence interval; DDDs, daily defined doses; OR, odds ratio.

### ACEI/ARB use and mortality in colorectal cancer patients

In colorectal cancer patients (Table 2), there was some evidence of a reduction in cancer-specific mortality in patients prescribed an ACEI (OR = 0.79, 95% CI 0.69, 0.92) which was little altered after adjustment for confounders (adjusted OR = 0.78, 95% CI 0.66, 0.92), but a clear dose response association was not apparent as there was little evidence of protective effects in those using more than 365 DDDs of ACEI (adjusted OR = 0.84 95% CI 0.68, 1.03). There was little evidence of an association between ARBs and cancer-specific mortality (adjusted OR = 0.82 95% CI 0.64, 1.07). Additional adjustment for stage little altered these estimates. Sensitivity analyses (Table 5) produced fairly consistent results for ARBs. Any protective association for ACEIs were attenuated when prescriptions in the year prior to death were removed (adjusted OR = 0.83 95% CI 0.67, 1.03) or when the analysis was restricted to users of any antihypertensive medication in the year prior to cancer diagnosis (adjusted OR = 0.83 95% CI 0.65, 1.07). In early stage colorectal cancer a more marked reduction in cancer specific mortality was observed with ACEI use (adjusted OR = 0.54 95% CI 0.38, 0.77) compared with later stage disease (adjusted OR = 0.86, 95% CI 0.68, 1.10; *P* for interaction = 0.03). In general, any protective associations were less marked when using the time varying covariate approach but other sensitivity analyses for ACEIs were similar to the main finding. No associations were observed between cancer-specific mortality and pre-diagnostic ACEI and ARB use (Table 3). Finally, there was little evidence of associations with all-cause mortality and ACEI (adjusted OR = 0.90, 95% CI 0.79, 1.02) or ARB (adjusted OR = 0.94, 95% CI 0.78, 1.13) use after cancer diagnosis (Table 4).

**Table 5:** Sensitivity analyses for association between ACEIs and ARBS and cancer specific mortality in colorectal cancer patients

	**Cancer specific deaths number**	**Controls number**	**OR (95% CI)**	***P***	**OR (95% CI)**	***P***
			**ACEI user versus non user**		**ARB user versus non user**	
**Colorectal cancer**						
Main analysis: diagnosis to six months prior to death^a^	1,093	5,231	0.76 (0.62, 0.93)	0.01	0.80 (0.59, 1.09)	0.16
Diagnosis to one year prior to death^b^	869	4,152	0.83 (0.67, 1.03)	0.10	0.89 (0.64, 1.25)	0.51
Restricted to users of any antihypertensive medication^c^ prior to cancer diagnosis^d^	427	1,934	0.83 (0.65, 1.07)	0.15	0.90 (0.63, 1.27)	0.54
Comparison group restricted to users of any antihypertensive in exposure period^c^	596	1,845	0.75 (0.61, 0.92)	0.01	0.80 (0.59, 1.10)	0.16
Additionally adjusting for other antihypertensives^e^	1,093	5,231	0.75 (0.61, 0.92)	0.01	0.80 (0.59, 1.09)	0.16
≥730 DDDs versus 0 DDDs (non-user)	1,093	5,231	0.73 (0.62, 1.00)	0.03	0.93 (0.64, 1.36)	0.72
Stage 1 and 2^f^	328	1,483	0.54 (0.38, 0.77)	0.001	0.80 (0.47, 1.37)	0.41
Stage 3 and 4^f^	740	3,185	0.86 (0.68, 1.10)	0.23	1.05 (0.72, 1.55)	0.79
Colon	649	3,134	0.84 (0.64, 1.09)	0.19	0.77 (0.52, 1.15)	0.21
Rectal	444	2,097	0.67 (0.49, 0.92)	0.01	0.91 (0.56, 1.50)	0.72
Males	638	3,078	0.80 (0.62, 1.03)	0.08	0.93 (0.62, 1.41)	0.74
Females	455	2,153	0.70 (0.49, 0.98)	0.04	0.64 (0.40, 1.03)	0.06
Pre-diagnostic use^g^	1,641	7,815	1.06 (0.89, 1.27)	0.51	0.86 (0.62, 1.18)	0.35
Time varying covariate analysis^h^	1,109	2,559				
≥1 DDDs versus 0 DDDs (non-user)			0.81 (0.69, 0.96)	0.01	0.86 (0.66, 1.12)	0.26
1 to365 DDDs versus 0 DDDs (non-user)			0.76 (0.59, 0.97)	0.03	0.64 (0.40, 1.03)	0.07
≥365 DDDs versus 0 DDDs (non-user)			0.85 (0.69, 1.03)	0.10	1.00 (0.73, 1.38)	0.99
≥1 DDDs versus 0 DDDs (non-user) in stage 1 and 2 patients			0.62 (0.46, 0.84)	0.002	0.65 (0.41, 1.05)	0.08
≥1 DDDs versus 0 DDDs (non-user) in stage 3 and 4 patients			0.91 (0.74, 1.10)	0.33	0.97 (0.70, 1.34)	0.83

^a^Except where otherwise stated analysis investigates medications from diagnosis to six months prior to death/index date and models include surgery (within six months of diagnosis), chemotherapy (within six months), radiotherapy (within six months), low dose aspirin (during exposure period), statins (during exposure period), comorbidities (pre-diagnosis or during exposure period, including myocardial infarction, cerebrovascular disease, congestive heart disease, chronic pulmonary disease, peripheral vascular disease, renal disease, peptic ulcer disease and diabetes), smoking (pre-diagnosis, with missing included as a category) and stage. ^b^Restricted to individuals surviving more than 1.5 years. ^c^Antihypertensive medications include beta-blockers, diuretics, vasodilator antihypertensive drugs, centrally acting antihypertensive drugs, alpha-adrenoceptor blocking drugs, ACEIs, ARBs, renin inhibitors and calcium channel blockers. ^d^Restricted to individuals with >1 year of records prior to cancer diagnosis and pre-diagnostic use considered antihypertensive use in that year, excluding deaths in the year after cancer diagnosis. ^e^Models include all variables in ^a^ and additionally include calcium channel blockers, diuretics and beta-blockers. ^f^Stratified analyses were conducted after re-matching controls to cases within strata, due to non-availability of matches overall numbers in subgroups may not be identical to numbers presented in Table 1. ^g^Restricted to individuals with >1 year of records prior to cancer diagnosis and pre-diagnostic use considered ACEI/ARB use in that year, not excluding deaths in the year after cancer diagnosis. ^h^Reported estimates are adjusted hazard ratios and 95% CI, model contains age at diagnosis, year of diagnosis, gender, site (colon or rectum), surgery (within six months of diagnosis), chemotherapy (within six months), radiotherapy (within six months), statins (during exposure period as a time varying covariate), comorbidities (pre-diagnosis including myocardial infarction, cerebrovascular disease, congestive heart disease, chronic pulmonary disease, peripheral vascular disease, renal disease, peptic ulcer disease and diabetes) and stage. ACEIs, angiotensin-converting enzyme inhibitors; ARBs, angiotensin II receptor blockers; CI, confidence interval; DDDs, daily defined doses; OR, odds ratio.

### ACEI/ARB use and mortality in prostate cancer patients

In prostate cancer patients (Table 2), there was no evidence of an increase in cancer-specific mortality in ACEI users (adjusted OR = 0.78, 95% CI 0.66, 0.92) or ARB users (adjusted OR = 0.79, 95% CI 0.61, 1.03). Additionally, adjusting for grade little altered the point estimates although the protective association with ACEIs was no longer significant (fully adjusted OR = 0.82 95% CI 0.67, 1.02). The associations for ARB use were fairly consistent across sensitivity analyses (Table 6). The protective association with ACEIs was more marked when restricting the analysis to antihypertensive users during the exposure period (adjusted OR = 0.78 95% CI 0.63, 0.96) and when adjusting for other antihypertensive use (adjusted OR = 0.74 95% CI 0.60, 0.92), but were attenuated in most other sensitivity analyses particularly when prescriptions in the year prior to death were removed (adjusted OR = 0.88 95% CI 0.70, 1.09) and in time-varying covariate analyses (adjusted hazard ratio (HR) = 0.90 95% CI 0.76, 1.07). There was little evidence of a reduction in all-cause mortality in ACEI users (adjusted OR = 0.91, 95% CI 0.82, 1.03) or ARB users (adjusted OR = 0.85, 95% CI 0.72, 1.01) after cancer diagnosis (Table 4).

**Table 6:** Sensitivity analyses for association between ACEIs and ARBS and cancer specific mortality in prostate cancer patients

	**Cancer specific deaths number**	**Controls number**	**OR (95% CI)**	***P***	**OR (95% CI)**	**P**
			**ACEI user versus non user**		**ARB user versus non user**	
**Prostate cancer**						
Main analysis: diagnosis to six months prior to death^a^	766	3,777	0.82 (0.67, 1.02)	0.07	0.82 (0.61, 1.11)	0.21
Diagnosis to one year prior to death^b^	682	3,360	0.88 (0.70, 1.09)	0.24	0.89 (0.64, 1.23)	0.48
Adjusting for Gleason score along with all variables in^a^ apart from grade	516	2,518	0.80 (0.62, 1.03)	0.09	0.89 (0.61, 1.31)	0.55
Restricted to users of any antihypertensive medication^c^ prior to cancer diagnosis^d^	399	1,925	0.80 (0.62, 1.04)	0.09	0.89 (0.62, 1.26)	0.51
Comparison group restricted to users of any antihypertensive in exposure period^c^	584	2,753	0.78 (0.63, 0.96)	0.02	0.80 (0.59, 1.08)	0.15
Additionally adjusting for other antihypertensives^e^	766	3,777	0.74 (0.60, 0.92)	0.01	0.75 (0.55, 1.02)	0.07
Additionally adjusting for hormone therapy any time after diagnosis^f^	766	3,777	0.82 (0.66, 1.01)	0.06	0.84 (0.62, 1.14)	0.27
≥730 DDDs versus 0 DDDs (non-user)	766	3,777	0.91 (0.70, 1.18)	0.49	0.84 (0.59, 1.21)	0.36
Pre-diagnostic use^g^	821	4,041	0.98 (0.78, 1.23)	0.87	1.14 (0.75, 1.75)	0.53
Time varying covariate analysis^h^	772	3,838				
≥1 DDDs versus 0 DDDs (non-user)			0.90 (0.76, 1.07)	0.24	0.95 (0.73, 1.23)	0.70
1 to 365 DDDs versus 0 DDDs (non-user)			0.87 (0.70, 1.08)	0.21	0.88 (0.62, 1.26)	0.49
≥365 DDDs versus 0 DDDs (non-user)			0.94 (0.76, 1.18)	0.60	1.03 (0.72, 1.48)	0.86

^a^Except where otherwise stated analysis investigates medications from diagnosis to six months prior to death/index date and models include surgery (within six months of diagnosis), chemotherapy (within six months), radiotherapy (within six months), low dose aspirin (during exposure period), statins (during exposure period), comorbidities (pre-diagnosis or during exposure period, including myocardial infarction, cerebrovascular disease, congestive heart disease, chronic pulmonary disease, peripheral vascular disease, renal disease, peptic ulcer disease and diabetes), smoking (pre-diagnosis, with missing included as a category), androgen therapy (within six months) and grade. ^b^Restricted to individuals surviving more than 1.5 years. ^c^Antihypertensive medications include beta-blockers, diuretics, vasodilator antihypertensive drugs, centrally acting antihypertensive drugs, alpha-adrenoceptor blocking drugs, ACEIs, ARBs, renin inhibitors, and calcium channel blockers. ^d^Restricted to individuals with >1 year of records prior to cancer diagnosis and pre-diagnostic use considered antihypertensive use in that year, excluding deaths in the year after cancer diagnosis.

^e^Models include all variables in ^a^and additionally include calcium channel blockers, diuretics and beta-blockers. ^f^Models include all variables in ^a^but androgen therapy was determined at any time after diagnosis in the exposure period. ^g^Restricted to individuals with >1 year of records prior to cancer diagnosis and pre-diagnostic use considered ACEI/ARB use in that year, not excluding deaths in the year after cancer diagnosis. ^h^Reported estimates are adjusted hazard ratios and 95% CIs models contain year of diagnosis, age at diagnosis, surgery (within six months of diagnosis), chemotherapy (within six months), radiotherapy (within six months), comorbidities (pre-diagnosis, including myocardial infarction, cerebrovascular disease, congestive heart disease, chronic pulmonary disease, peripheral vascular disease, renal disease, peptic ulcer disease and diabetes), statin (as a time varying covariate) and grade. ACEIs, angiotensin-converting enzyme inhibitors; ARBs, angiotensin II receptor blockers; CI, confidence interval; DDDs, daily defined doses; OR, odds ratio.

## Discussion

Overall, we found no evidence of increased risks of cancer-specific or all-cause mortality in breast, colorectal or prostate cancer patients using ACEIs or ARBs after cancer diagnosis. There was some evidence of reductions in the risk of cancer-specific mortality in colorectal and prostate cancer patients using ACEIs, but any protective effects were weak in magnitude, were inconsistent across sensitivity analyses and were not *a priori* stated and, therefore, are difficult to interpret.

### Strengths and weaknesses

The main strengths of our study were the large size (including over 20,000 cancer patients and 4,000 cancer-specific deaths) and the long duration of follow-up (up to 13 years) which provided the ability to detect relatively weak effects and to report narrow CIs which rule out relatively small increases in risk. Our study used GP prescribed drug information which captures almost all ACEI and ARB use (which are only available by prescription in the UK), eliminates the potential for recall bias incurred by self–report and allows detailed temporal associations to be explored. Consequently, our data reflect GP prescriptions rather than drug consumption but analyses of multiple prescriptions generally found similar results, suggesting compliance may not impact our results greatly. As with all observational studies we cannot exclude residual or unknown confounding. We had robust data on surgery, radiotherapy, chemotherapy, hormone therapy, comorbidities and importantly stage for colorectal and breast and Gleason score for prostate cancer, but we had limited data on smoking and alcohol intake and no information on socioeconomic status.

### Comparison with previous studies

In prostate and colorectal cancer our study is the first epidemiological study to investigate ARBs separately and cancer progression and only three studies have investigated ACEI and cancer progression, one [17] in prostate cancer patients and two [15,16] in colorectal cancer patients. An earlier ACEI study in 62 prostate cancer patients after radical prostatectomy [17] observed a reduced rate of biochemical recurrence in ACEI users compared with non-users (3/32 versus 10/30). An earlier ACEI study in 55 stage 2 colorectal cancer patients [15] observed a reduction in the risk of distant metastasis with frequent ACEI use prior to diagnosis (adjusted OR = 0.22) and a more recent study in 262 stage 3 and 4 cancer patients did not present an estimate for ACEI or ARB use separately but did present a reduced risk of mortality in patients simultaneously using beta-blockers and ACEIs or ARBs (HR = 0.50 95% CI 0.29, 0.85).

Five independent epidemiological studies [13,14,18-20] have previously investigated ACEI and cancer-specific mortality in breast cancer patients but only two have previously investigated ARB use separately. A recent large Danish study [13] demonstrated a small but non-significant increase in cancer recurrence with ACEI use but no association with ARB use (HR = 1.2 95% CI 0.97, 1.4 and HR = 1.1 95% CI 0.85, 1.3, respectively). Another recent US study [14], from the same cohort as an earlier study [34], observed no association between either ACEI use (HR = 1.07 95% CI 0.65, 1.77) or ARB use (HR = 0.41 95% CI 0.15, 1.13) and cancer-specific mortality. An earlier smaller study [18], including 174 breast cancer-specific deaths in 1,779 breast cancer patients, observed no association with simultaneous ACEI and beta-blocker use and when investigating ACEI users exclusively observed a marked increase in cancer recurrence (HR = 1.56 95% CI 1.02, 2.39) but little evidence of an increase in breast cancer-specific mortality (HR = 1.27 95% CI 0.74, 2.19). One study observed little evidence of association for self-reported ACEI use (HR = 0.89 95% CI 0.60, 1.32) [20]. Finally another study observed a marked reduction in cancer recurrence in 168 stage 2 or 3 breast cancer patients using ACEI or ARBs after diagnosis (HR = 0.57 95% CI 0.37, 0.89, *P* =0.01) [19], but this estimate may have incurred some immortal time bias [29] as individuals using ACEIs or ARBs after diagnosis were considered users from diagnosis in the analysis so in the period from diagnosis to medication use they could not have died.

Our study observed some protective effects of ACEIs, particularly in early stage colorectal cancer patients and prostate cancer patients, which, if real, would support the theory that ACEIs have a role in cancer therapy [35,36]. Components of the renin-angiotensin system (RAS) are expressed in various cancer sites [37] and may contribute to processes important for cancer progression including cell proliferation and apoptosis. *In vitro* and animal studies have shown that both ACEIs and ARBs can suppress cell proliferation and tumor/metastasis growth in various cancers including breast [38] and colorectal [39]. Angiogenesis also appears to be an important process by which the RAS system exerts pro-tumor effects and ACEIs and ARBs reduce the expression of vascular endothelial growth factor (VEGF) and other angiogenic factors in both cell lines [38] and animal models [40]. However, the protective effects observed in colorectal and prostate cancer patients should be interpreted cautiously because, as previously stated, the effects were weak, inconsistent and not *a priori* stated.

## Conclusions

In conclusion, concerns about the safety of ACEIs and ARBs in cancer patients have been raised by trial data showing increases in fatal cancers in ARB users [4] and observational data showing increases in breast cancer recurrence rates in ACEI users [18]. In contrast, our study provides no evidence of increased risks of cancer-specific mortality in users of ACEIs or ARBs with breast, prostate or colorectal cancer and suggests that these medications are safe in patients diagnosed with these common cancers.

## Competing interests

The authors declare that they have no competing interests.

## Authors’ contributions

Study concept and design: CRC, CH, LJM. Acquisition of data: CRC, UCM, CH, MMC, LJM. Analysis and interpretation of data: all authors. Drafting of the manuscript: CRC. Critical revision of the manuscript for important intellectual content: all authors. Statistical analysis: CRC, UCM, BMH. Obtained funding: CRC, CH, LJM. All authors read and approved the final manuscript.

## Pre-publication history

The pre-publication history for this paper can be accessed here:

http://www.biomedcentral.com/1741-7015/12/28/prepub
